# T Cell Interactions in Mycobacterial Granulomas: Non-Specific T Cells Regulate Mycobacteria-Specific T Cells in Granulomatous Lesions

**DOI:** 10.3390/cells10123285

**Published:** 2021-11-24

**Authors:** Dominic O. Co, Laura H. Hogan, Jozsef Karman, Melinda Herbath, Zsuzsanna Fabry, Matyas Sandor

**Affiliations:** 1 Department of Pediatrics, School of Medicine and Public Health, University of Wisconsin-Madison, Madison, WI 53705, USA; doco@wisc.edu; 2 The Institute for Clinical and Translational Research, University of Wisconsin-Madison, Madison, WI 53705, USA; lhh@medicine.wisc.edu; 3 Cambridge Research Center, Abbvie, Inc., Cambridge, MA 02139, USA; jkarman@gmail.com; 4 Department of Pathology, School of Medicine and Public Health, University of Wisconsin-Madison, Madison, WI 53705, USA; herbath@wisc.edu (M.H.); zfabry@wisc.edu (Z.F.)

**Keywords:** granuloma, T cell, mycobacterium

## Abstract

Infections with pathogenic mycobacteria are controlled by the formation of a unique structure known as a granuloma. The granuloma represents a host–pathogen interface where bacteria are killed and confined by the host response, but also where bacteria persist. Previous work has demonstrated that the T cell repertoire is heterogenous even at the single granuloma level. However, further work using pigeon cytochrome C (PCC) epitope-tagged BCG (PCC-BCG) and PCC-specific 5CC7 RAG^−/−^ TCR transgenic (Tg) mice has demonstrated that a monoclonal T cell population is able to control infection. At the chronic stage of infection, granuloma-infiltrating T cells remain highly activated in wild-type mice, while T cells in the monoclonal T cell mice are anergic. We hypothesized that addition of an acutely activated non-specific T cell to the monoclonal T cell system could recapitulate the wild-type phenotype. Here we report that activated non-specific T cells have access to the granuloma and deliver a set of cytokines and chemokines to the lesions. Strikingly, non-specific T cells rescue BCG-specific T cells from anergy and enhance the function of BCG-specific T cells in the granuloma in the chronic phase of infection when bacterial antigen load is low. In addition, we find that these same non-specific T cells have an inhibitory effect on systemic BCG-specific T cells. Taken together, these data suggest that T cells non-specific for granuloma-inducing agents can alter the function of granuloma-specific T cells and have important roles in mycobacterial immunity and other granulomatous disorders.

## 1. Introduction

Approximately, one-third of the world’s population is infected with Mycobacterium tuberculosis (Mtb) earning it the distinction of the “world’s most successful pathogen.” [1]. Despite a vigorous immune response, chronic infection persists, and reactivation or reinfection can occur. The primary cause of morbidity and mortality in tuberculosis is the failure of the host response to control reactivation during the chronic phase of infection. Thus, an understanding of the host response to Mtb during latent infection is critical to controlling the disease. In this work, we describe how activated T cells lacking specificity for the infection can contribute to protection during chronic infection.

CD4^+^ T cells have a central role in protection against mycobacterial diseases by orchestrating the formation of a delayed-type hypersensitivity site, the granuloma [2,3,4]. The granuloma is a host–pathogen interface where the host immune response controls bacteria, but also where bacteria persist. Although the granuloma environment may hold the key to bacterial persistence and the absence of sterilizing immunity, knowledge of the biology of this compartment remains scarce. Mice lacking CD4^+^ T cells due to deficiency in recombinase activating gene (RAG), TCR β chain [5], CD4, or MHC class II [6,7] succumb to infections with mycobacteria. Furthermore, adoptive transfer of CD4^+^ T cells to deficient hosts confers protection [8] and promotes granuloma formation [9]. CD4^+^ T cells participate in every aspect of granuloma formation [10] and can mediate protection through a variety of mechanisms. The cytokines TNF [11,12,13] and IFN-γ [14,15] are key factors in protection against mycobacteria, though many other factors are known to play a role.

Control of bacterial numbers during the chronic phase of infection depends upon a balance between bacterial proliferation and T cell immunity. Declining CD4^+^ T cell counts in the late stages of HIV/AIDS results in the reactivation of previously latent mycobacterial infections [16]. In mouse models, antibody depletion of CD4^+^ T cells during the chronic phase of infection also results in reactivation [17]. Previous work from our laboratory and other groups has demonstrated that the T cell repertoire is broad even at the level of the single granuloma and differs from lesion to lesion [9,18,19]. Despite this broad repertoire, a single monoclonal BCG-specific T cell population is sufficient to mediate protective granuloma formation [9]. Although protection is equivalent under the conditions studied thus far, we have noted differences in the activation phenotype of CD4^+^ T cells in wild-type mice infected with BCG as compared with mice possessing monoclonal populations of CD4^+^ T cells specific for BCG. In this work, our goal was to determine whether a two T cell system could recapitulate some of the properties of infection of wild-type mice.

In genetically intact or wild-type mice, the diversity of the normal T cell repertoire makes it difficult to distinguish pathogen-specific T cells from non-specific T cells. To test how non-specific T cells contribute to granulomatous lesions, we constructed a model system in which mycobacteria-specific and non-specific T cells were specified as two monoclonal T cell populations in contrast to the millions of specificities present in the normal repertoire. PCC-specific CD4^+^ TCR Tg 5CC7 RAG^−/−^ mice were infected with recombinant *M. bovis* strain bacille Calmette–Guérin (BCG) expressing PCC (PCC-BCG). These mice were adoptively transferred with T cells from the conalbumin (CA) specific CD4^+^ TCR Tg D10 RAG^−/−^ as sentinels for a BCG-non-specific T cell population. We demonstrate that BCG-non-specific D10 T cells, when activated, have access to the granulomatous inflammatory site. Additionally, these non-specific T cells affect the function of BCG-specific T cells in the granuloma. These non-specific T cells also contribute cytokines and influence macrophage activation in the granuloma. These data suggest a role for activated non-specific T cells in boosting the activity of local antigen-specific T cells and in directly affecting the antimicrobial function of the granuloma. Our data indicate that non-specific and specific T cells can cooperate to control bacteria at the site of infection.

## 2. Materials and Methods

### 2.1. Mice

5CC7 RAG2^−/−^ mice [20], specific for PCC residues 88–104 in the context of I-E^k^, were purchased on the B10.A background from Taconic Farms Emerging Models Program (Tarrytown, NY, USA) and bred onto the B10.BR RAG1^−/−^ background. D10 RAG1^−/−^ mice [21] specific for CA residues 121–136 in the context of I-A^k^ were maintained on the B10.BR RAG^−/−^ background. Mice for all experiments were used at 6–10 weeks of age. Mice were bred and housed at the University of Wisconsin Animal Care Unit (Madison, WI, USA) under specific pathogen free (SPF) conditions in filter top cages with autoclaved cages, water, bedding and feed according to the guidelines of the Institutional Animal Care and Use Committee (IACUC).

### 2.2. Infections

Creation of PCC-BCG from BCG Pasteur strain (Staten Serum Institut, Copenhagen, Denmark) was described previously [9]. Growth and preparation of frozen stocks for infection were as described [9]. For infection, 7 × 10^6^ cfu of BCG were injected intraperitoneally (i.p.).

### 2.3. Testing Transgenic T Cells for Reactivity to PCC-BCG Antigens

Frozen stocks of PCC-BCG were sonicated with three pulses at high power in a Sonicator ^®^ ultrasonic processor (Heat Systems, Newtown, CT, USA) to disrupt the cell wall. The BCG sonicate was then centrifuged at 14,000 rpm in an Eppendorf centrifuge to separate a “lysate” fraction, and the “pellet” fraction was resuspended in PBS. D10 spleen cells were plated at 10^6^ per well in a 96 well plate in 200 μL complete RPMI 1640 plus 10% FBS (cRPMI10) with either 10 μL or 50 μL of BCG lysate or pellet. After 72 h of culture, cells were harvested, washed and analyzed for their expression of activation markers by flow cytometry.

### 2.4. Cell Isolation and Flow Cytometry

Isolation of splenocytes and granuloma-infiltrating cells was performed as described previously [9] to produce single cell suspensions. Note that livers for isolating granuloma infiltrating cells so granuloma data represent granuloma infiltrating cells from two to four mice. Cells (10^6^) were incubated for 30 min on ice with saturating concentrations of labeled antibodies and 40 μg/mL unlabeled 2.4G2 mAb to block binding to Fc receptors. Washed samples were analyzed on a Becton Dickinson FACSCalibur (Mountain View, CA, USA). Flow cytometric data was analyzed using FlowJo software version 4.6.1 (Treestar, Ashland, OR, USA). Fluorochrome-labeled antibodies against CD4 (RM5-4), LFA-1 (7D4), CD45RB (16A), and TCR Vβ3 (KJ25) were purchased from Pharmingen (Mountain View, CA, USA). Hybridomas for antibodies against TCR Vβ3 (KJ25), I-A^k^ (10-2.16), CD11b (Mac-1), Vβ8 (F23.1), were purchased from American Type Culture Collection (Manassas, VA, USA) and hybridoma for the antibody against D10 TCR (3D3) was a generous gift of Derek Sant’Angelo (Memorial Sloan Kettering Cancer Center, New York, NY, USA) [22]. These antibodies were produced from hybridomas and labeled with NHS-biotin (Sigma, St. Louis, MO, USA), FITC (Sigma, St. Louis, MO, USA), or CY5 (Amersham Pharmacia Biotech, Piscataway, NJ, USA) according to package instructions.

### 2.5. Adoptive Transfer and Immunization

Spleen cell suspensions prepared from D10 RAG^−/−^ equivalent to 1 × 10^6^ CD4^+^ Vβ8^+^ T cells were adoptively transferred to PCC-BCG infected 5CC7 RAG^−/−^ mice at 5 weeks post-infection. The following day, mice were immunized subcutaneously (s.c.) with either 100 μg conalbumin or PBS (vehicle control) emulsified with incomplete Freund’s adjuvant (IFA).

### 2.6. Immunofluorescence

All incubations were performed at room temperature unless otherwise stated. Five-μm-thick cryosections were cut from frozen O.C.T. embedded tissues and fixed for 30 min in 4% PFA in PBS. Sections were then washed three times with PBS and outlined with a Pap pen. Sections were blocked for 30 min with 1% BSA and 40 μg/mL 2.4G2 antibody to block FcR binding and stained for 30 min with Alexa 568-labeled KJ25 (anti-Vβ3) and FITC-labeled F23.1 (anti-Vβ8) in the presence of 1% BSA and 40 μg/mL 2.4G2. Unbound antibody was washed away by three washes in PBS and sections were coverslipped with Gel/Mount (Biomeda, Goleta, CA, USA). Slides were viewed on an Olympus IX-70 fluorescent microscope equipped with an Optronics DEI 750 digital camera. Images were acquired with LaserSharp Acquisition Software and analyzed with BioRad Confocal Assistant.

### 2.7. Histopathology

Tissue was fixed in 10% neutral buffered formalin and processed for paraffin embedding by standard methods. 5 μm thick sections were stained with H&E for tissue morphology and by the Ziehl–Neelsen method to identify acid-fast bacilli (AFB). To quantitate granuloma lesion size, digital images of H&E stained sections were acquired at 400× total magnification and the granuloma area was determined by outlining each lesion in the Scion Image program version 1.62c (National Institutes of Health, Bethesda, MD, USA). Quantitation of granuloma burden was performed by counting the number of liver granulomas per field at 100× total magnification. To quantitate bacterial load, the number of AFB per lesion were counted at 1000× total magnification on Ziehl–Neelsen stained slides.

### 2.8. Multiplex Cytokine Assay

Spleen or granuloma cells (10^6^ per well of a 96-well plate) were cultured in triplicate with 100 μg/mL PCC or CA in cRPMI10; 72 h later, culture supernatants were harvested and stored at −80 °C. Frozen supernatants were assayed on the 22-plex Mouse Cytokine and Chemokine cytometric bead array as a service by LINCO Research (St. Charles, MO, USA). Intra-assay and inter-assay variances were less than 10% and 20%, respectively.

## 3. Results

**Figure 1:**
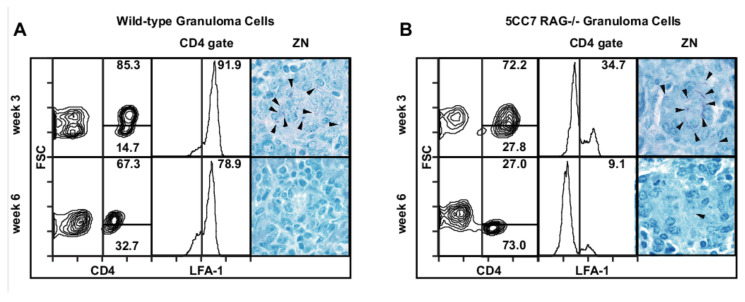
Wild-type mice and 5CC7 RAG^−/−^ mice both form protective granulomas after PCC-BCG infection but 5CC7 T cells show lower levels of activation. (**A**), Wild-type mice were infected intraperitoneally (i.p.) with BCG and stained for CD4 and LFA-1 at three and six weeks post-infection. Numbers in left panels represent percentage of CD4^+^ that are large (blasting). Middle panel is gated on CD4^+^ T cells as shown in the left panel and numbers represent the percent of CD4^+^ T cells that are LFA-1^high^. The right panel shows Ziehl–Neelsen staining for acid-fast bacilli (AFB) indicated by arrowheads. (**B**), 5CC7 RAG^−/−^ mice were infected i.p. with PCC-BCG and stained for CD4 and LFA-1 at three and six weeks postinfection. Numbers in leftmost panels represent percentage of CD4^+^ that are large/blasting. Second panel from left is gated on CD4^+^ T cells as shown in the leftmost panel and numbers represent the percent of CD4^+^ T cells that are LFA-1^high^. Rightmost panel shows Ziehl–Neelsen staining for acid-fast bacilli (AFB) indicated by arrowheads. Figure representative of three independent experiments with 3 mice per group.

In 5CC7 RAG^−/−^ mice chronically infected with PCC-BCG, 5CC7 T cells acquire a resting phenotype. Previous work has demonstrated that 5CC7 RAG^−/−^ mice expressing a monoclonal T cell population specific for PCC-BCG control bacteria similarly to wild-type mice possessing the normal repertoire of T cells [9]. (Figure 1A,B, rightmost panels) shows that both wild-type and 5CC7 RAG^−/−^ mice have high levels of acid-fast bacilli (AFB) at three weeks but low levels of AFB during the chronic stage of infection at six weeks. However, the activation state of granuloma-infiltrating T cells in infected 5CC7 RAG^−/−^ mice as measured by cell size and by cell surface levels of LFA-1 decreases to a much greater extent during chronic infection than the activation state of granuloma-infiltrating T cells in wild-type mice (Figure 1A,B, first and second panel from left). The repertoire of T cells in wild-type chronic granulomas is broad and contains both pathogen-specific T cells and pathogen-non-specific T cells [9]. To test the hypothesis that non-specific T cells contribute to the T cell activation phenotype in wild-type granulomas, we introduced a BCG-non-specific T cell population from CA-specific, D10 RAG^−/−^ TCR Tg to the monoclonal 5CC7 T cell system.

### 3.1. D10 T Cells Are Not Activated by PCC-BCG

**Figure 2:**
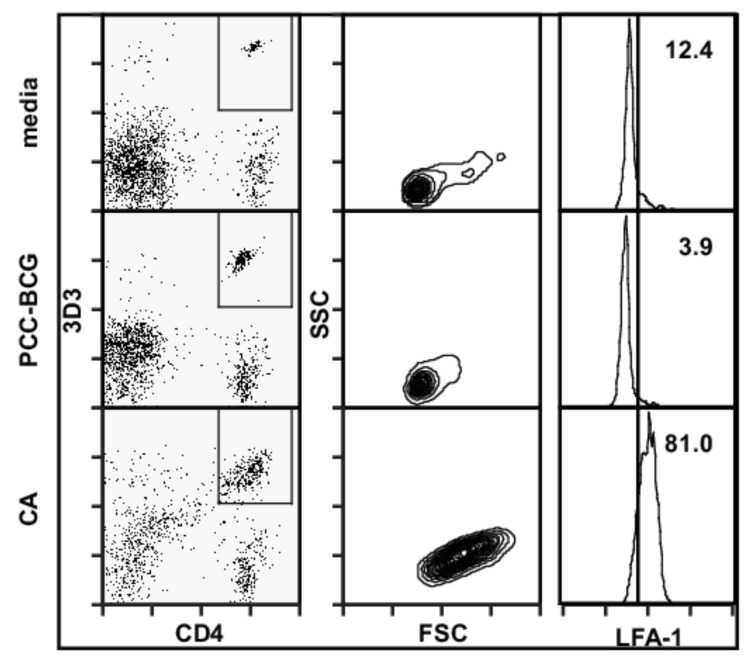
PCC-BCG does not activate D10 T cells in vitro. D10 spleen cells were cultured with PCC-BCG sonicates for 3 days in cRPMI10. D10 spleen cells were cultured with media alone (**top**) or with 100 μg/mL CA (**bottom**) as negative and positive controls, respectively. Cells were harvested and stained with the D10 clonotypic antibody 3D3 and antibodies to CD4 and the activation marker LFA-1. Leftmost plots show CD4 and 3D3 expression on lymphocyte-gated cells. Middle plots show forward (FSC) and side scatter (SSC) on CD4^+^ 3D3^+^ T cells, and right plots show level of LFA-1 expression on CD4^+^ 3D3^+^ T cells. These data representative of three independent experiments.

**Figure 3:**
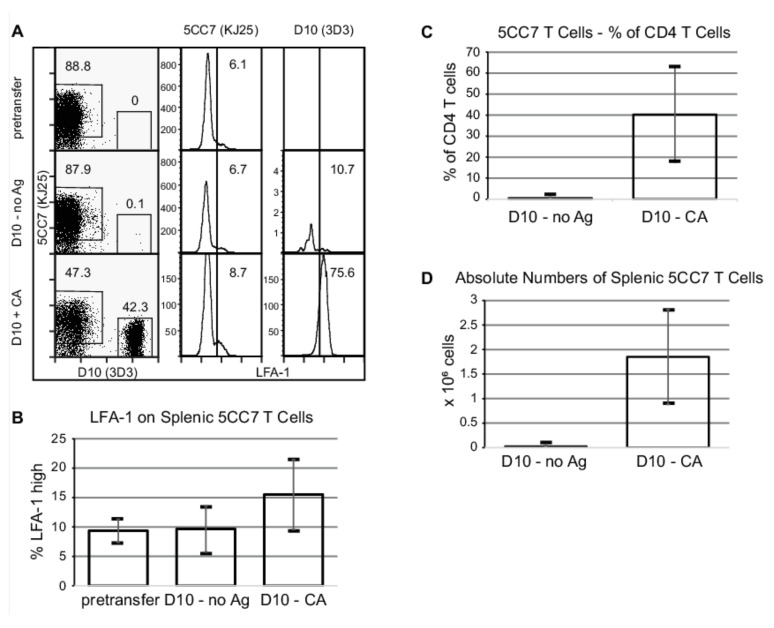
Immunization results in a robust expansion of expansion and activation of CA-specific T cells. 5CC7 RAG^−/−^ mice were infected with PCC-BCG. At five weeks of infection, mice were transferred with spleen cells from D10 RAG^−/−^ mice. The next day, mice were immunized subcutaneously with CA or PBS as a control. One week after immunization, spleen cells were isolated and stained for flow cytometry. (**A**), Left panel is gated on CD4^+^ lymphocytes. KJ25 (middle) and 3D3 (right) panels were gated as shown in the left panel and numbers represent percent of gated cells expressing high levels of LFA-1. (**B**), Quantification of the number of splenic 5CC7 T cells that show high levels of the activation marker LFA-1. (**C**), Expansion of the D10 T cells quantified as % of CD4 T cell compartment. (**D**), Expansion of D10 T cells quantified as absolute numbers. In panels B–D, bars represent mean and error bars represent standard deviation. Data is representative of three independent experiments with 2–4 mice per group.

To assess for reactivity of D10 T cells to PCC-BCG antigens, a BLAST search of the Mtb and BCG genomes for DNA sequences encoding the CA 121–136 epitope was performed and yielded no sequences with significant homology. D10 T cells cultured in vitro with PCC-BCG exhibited no evidence of blasting (Figure 2, second column from the left) or elevation of LFA-1 expression (Figure 2, third column from the left). Finally, D10 T cells adoptively transferred to PCC-BCG-infected mice demonstrated no evidence of expansion or activation (Figure 3, second row) after one week, while immunization with CA Ag resulted in robust expansion and activation (Figure 3, third row). These data demonstrate that D10 T cells have no reactivity for PCC-BCG antigens in vitro or in vivo, and that D10 T cells are a useful sentinel population for BCG-non-specific T cells. Thus, 5CC7 RAG^−/−^ mice adoptively transferred with D10 cells represent a two T cell system in which one monoclonal T cell population is specific for BCG, while the other is not specific for BCG.

### 3.2. Acutely Activated BCG-Non-Specific T Cells Have Access to the Granuloma

**Figure 4:**
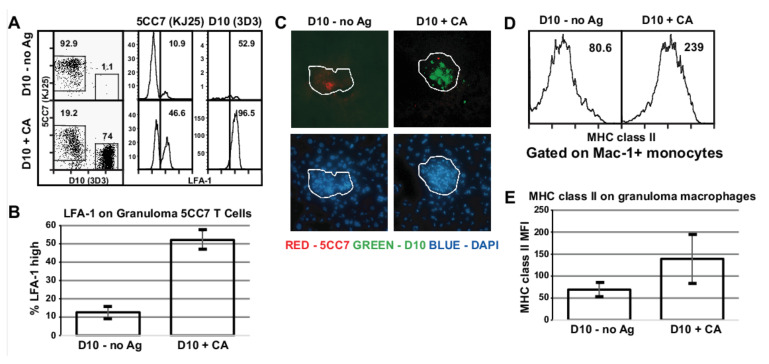
Acutely activated D10 T cells home to granulomas and activate granuloma-infiltrating 5CC7 T cells and macrophages. (**A**), Granuloma cells were isolated from mice treated as described in the legend to Figure 3 and stained for flow cytometry. Left plots are gated on CD4^+^ lymphocytes. 5CC7 (middle) and D10 (right) panels were gated as shown in the left panel and numbers represent percent LFA-1^high^ cells according to the gate shown. (**B**), Quantification of the percentage of LFA-1^high^ 5CC7 T cells. Bars represent mean and error bars represent standard deviation. (**C**), Liver tissue was embedded in O.C.T. and processed for frozen sections. Sections were stained with Alexa 488-anti-Vβ8 (green, left upper panel), Alexa 568-anti-Vβ3 (red, right upper panel) and DAPI (blue, lower panels) as a nuclear counterstain to outline granulomas. Fluorescent antibody stains and DAPI stains are displayed separately for clarity. White lines in panel C represent granuloma lesion outline. (**D**), Granuloma cells were stained for cell surface expression of I-A^k^ on Mac-1^+^ monocytes. Numbers represent mean fluorescence intensity. (**E**), Quantification of MFI changes from panel (**D**). Bars represent mean of MFI with error bars showing standard deviation. Data representative of three independent experiments with 2–4 mice per group.

We used this two T cell network model to test the hypothesis that T cells lacking specificity for granuloma antigens can accumulate in the granuloma. 5CC7 RAG^−/−^ mice were infected with PCC-BCG i.p. to induce liver granulomas. At 5 weeks post-infection, when granuloma formation is chronic, mice were transferred with D10 RAG^−/−^ spleen cells equivalent to 10^6^ CD4^+^ cells and immunized s.c. with either CA antigen or PBS. One week post-transfer, spleen and granuloma infiltrating cells were isolated and stained to distinguish the two transgenic T cell populations by flow cytometry. Previous work using RAG^−/−^ mice infected with PCC-BCG and adoptively transferred with PCC-specific T cells demonstrated that one week is sufficient to allow protective granuloma formation [9]. Immunization results in a robust activation as measured by LFA-1 expression (Figure 3B). In addition, immunization resulted in approximately 80-fold expansion of transferred D10 T cells in the spleen (mean from three independent experiments of 2.23 × 10^4^ splenic D10 T cells in the PBS-immunized mice vs. 1.85 × 10^6^ D10 T cells in the CA-immunized mice; Figure 3C,D). In the granuloma, activation of D10 T cells by antigen allowed them to accumulate in granulomas and outnumber 5CC7 (PCC-BCG-specific) T cells by a ratio of approximately 5:1 (Figure 4A,B). As in the spleen of CA-immunized BCG-infected mice, granuloma-infiltrating D10 T cells express high levels of LFA-1. These data suggest that activated non-specific T cells can upregulate the necessary factors, such as adhesion molecules for accumulation at the BCG inflammatory site. In addition, they suggest that if activated T cells dominate systemically, they dominate the local inflammatory site as well.

To confirm the localization of D10 T cells to the granuloma, frozen sections of liver tissue from these mice were stained with antibodies specific for 5CC7 (red) and D10 (green) T cell receptors and counterstained with DAPI (blue) to identify lesions. As shown in Figure 4C, granulomatous lesions in nonactivated mice contain only PCC-specific cells, whereas in activated mice, the CA-specific T cells dominate the lesions. In addition, the CA-specific T cells are confined largely to the granulomatous lesions.

### 3.3. Acute Activation of Local Antigen Non-Specific T Cells Increases the Activation of Granuloma Antigen Specific T Cells

The large proportion of CA-specific T cells in the PCC-BCG-induced granulomas of activated mice suggested that these activated cells might have an effect on the ongoing immune response to PCC-BCG. Cytokines produced during expansion of antigen-specific T cells can induce activation of bystander T cells [23]. At this chronic stage in PCC-BCG infection, splenic 5CC7 cells have already downregulated LFA-1 (Figure 3A, middle column). As expected, LFA-1 levels on 5CC7 T cells are higher in the granuloma than in the spleen since granulomas accumulate activated T cells. Activation of transferred D10 T cells shows a trend towards increased activation of the BCG-specific 5CC7 T cells, but this is did not reach statistical significance (Figure 3A, middle column, and quantified in Figure 3B). At the same time, in the granulomas of CA-immunized mice, there was a nearly fivefold increase in the proportion of activated 5CC7 T cells as measured by high cell surface levels of LFA-1 (Figure 4A, middle column, and quantified in Figure 4B) and by cell size (Figure S1). Remarkably, these results suggest that activation of non-specific T cell populations can increase the activation state of granuloma antigen-specific T cells. As discussed later (Figure 4D), activated non-specific T cells alter the functional properties of granuloma-infiltrating BCG-specific T cells as well.

### 3.4. CA Restimulation of Granuloma-Infiltrating D10 T Cells from CA-Immunized Mice Elicits a Profile of Cytokines That Can Contribute to Protection against Mycobacteria

Given the access of D10 T cells to BCG granulomas in mice immunized with CA/IFA, we examined the cytokine signature of spleen and granuloma cells from these mice in response to in vitro restimulation with CA using a cytometric bead array (CBA, Table 1). CA restimulation induced high levels of IL-2 production from granuloma cells and even higher levels from spleen cells. In addition, spleen and granuloma cells restimulated with CA secreted high levels of Th1 cytokines IFN-γ and TNF, while secreting lower levels of Th2 cytokines IL-4, IL-5, IL-9, and IL-13. This is important as it suggests that homing of D10 T cells to granulomas increases the levels of cytokines that are known to be important in protection against mycobacterial infection. With regard to chemokine secretion, granuloma cells secreted higher constitutive levels of MCP-1, RANTES, and IP-10, which attract monocytes and T cells, with a preference for antigen-experienced T cells. Again, this data suggests that the presence of activated non-specific T cells can augment the recruitment of cells to granulomas. While the range of cytokines produced by CA-restimulated spleen and granuloma cells in CA-immunized mice is similar, there are differences between the two sites. Most notably, granuloma-infiltrating cells produce much more MIP-1α than spleen cells, suggesting that granuloma-infiltrating non-specific T cells have a cytokine profile better suited to the granuloma environment and distinct from the profile of systemic non-specific T cells. Taken together these data indicate that activated non-specific T cells can directly contribute to cytokine secretion and cellular recruitment in granulomas.

### 3.5. PCC-Stimulated Cytokine Secretion by Granuloma Infiltrating Cells Is Upregulated by Recruited Activated BCG-Non-Specific T Cells

Table 2 illustrates the profile of cytokines secreted by spleen and granuloma cells in response to PCC and highlights the effect of activation of D10 T cells by CA immunization on PCC-stimulated cytokine secretion. In contrast to the spleen, 5CC7 T cells in chronic granulomas are anergic as they do not secrete IL-2 or IFN-γ in response to restimulation with cognate antigen (PCC). The most important finding is that the presence of activated D10 T cells in granulomas alters not only the activation phenotype of 5CC7 T cells (Figure 4A), but also their ability to secrete IL-2 and IFN-γ (Table 2) indicating rescue from anergy. In addition, recruitment of acutely activated D10 T cells also leads to higher constitutive levels of TNF, MCP, RANTES, IP-10, KC, GM-CSF and G-CSF. Overall, these data indicate that activation of non-specific (D10) T cells by immunization partially reverses anergy of BCG-specific 5CC7 T cells in the granuloma and results in a Th1-biased PCC-induced cytokine secretion profile. In addition, activation of non-specific T cells leads to the secretion of chemokines that can recruit more macrophages and activated T cells. Taken together, these data suggest that mycobacteria-non-specific T cells alter and potentially augment the function of mycobacteria-specific T cells in chronic granulomas.

### 3.6. Activation of Non-Specific T Cells Suppresses Cytokine Secretion by BCG-Specific Spleen Cells

Interestingly, while activation of non-specific T cells by CA immunization results in increased cytokine secretion by granuloma cells in response to in vitro PCC restimulation, CA immunization leads to decreased cytokine secretion by splenic cells in response to PCC (Table 2, left half). In general, spleen cells from non-transferred and D10-transferred, PBS-immunized mice that are restimulated with PCC exhibit a Th1-biased cytokine profile characterized by high levels of IFN-γ and TNF and low levels of IL-4, IL-5, IL-9 and IL-13. This suggests that while 5CC7 T cells in the local BCG inflammatory site are anergic, systemic T cells are not. In contrast, spleen cells from D10-transferred, CA-immunized mice restimulated with PCC secreted approximately tenfold less IL-2, IFN-γ and MIP-1α than PCC-restimulated spleen cells from D10-transferred, PBS-immunized mice. The magnitude of these changes suggests that D10 T cells have an active suppressive effect on 5CC7 T cells in the spleen. This observation suggests that when both cells are activated, they compete with each other in lymphoid organs, whereas they seem to cooperate and enhance each other’s function at the local inflammatory site.

### 3.7. Accumulation of Activated D10 T Cells Results in Increased Granuloma Macrophage Activation

To assess the in vivo effect of IFN-γ on granuloma function, we measured cell surface expression of I-A^k^ (Figure 4D) as an indicator of macrophage activation. Consistent with elevated levels of both 5CC7 and D10 activation as well as increased IFN-γ secretion in CA-immunized mice, MHC class II was moderately elevated on splenic macrophages and significantly elevated on granuloma infiltrating macrophages from CA-immunized mice relative to PBS-immunized mice. These results suggest that acutely activated non-specific T cells can indirectly contribute to granuloma function by rescuing antigen-specific T cells from anergy. In addition, activated non-specific CD4^+^ T cells can directly participate in effector functions in the granuloma, including IFN-γ production to effect bacterial killing and chemokine production to aid in recruitment of additional macrophages and T cells to the granuloma.

### 3.8. Effect of Non-Specific T Cells on Granuloma Structure and Function

**Figure 5:**
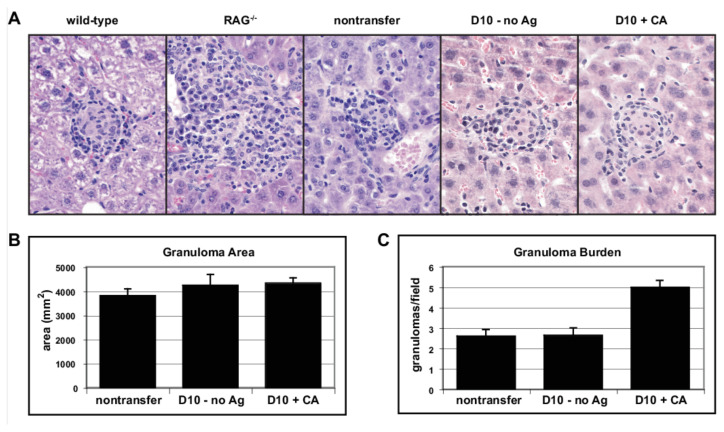
Immunization with CA does not alter granuloma size and structure. (**A**), Formalin fixed liver tissue from BCG-infected C57BL/6, B10.BR RAG^−/−^, 5CC7 RAG^−/−^, 5CC7 RAG^−/−^ + D10 T cells, and 5CC7 RAG^−/−^ + D10 T cells + CA (left to right) was embedded in paraffin and sections stained with H&E. (**B**), Digital images were acquired of H&E stained lesions at 400× total magnification. Lesions were outlined to measure the granuloma area in the Scion Image program (NIH). (**C**), Number of liver granulomas per field at 100× total magnification was counted. Error bars represent SEM. Data representative of three independent experiments with 2–4 mice per group.

Given the increased numbers of activated effector cells present in the CA-immunized two T cell granuloma, we questioned if this altered cell population would lead to increased control of BCG infection or altered granuloma morphology. Figure 5A presents representative micrographs from the three experimental groups alongside micrographs from wild-type C57BL/6 and RAG^−/−^ mice for comparison. BCG-induced granulomas formed in the mice possessing either one or two T cell specificities appear similar to wild-type granulomas. Quantitation of granuloma area using digital images revealed no statistically significant differences in granuloma size between the different groups (Figure 5B). Quantitation of the number of granulomas per unit area (granuloma burden, Figure 5C) revealed a modest increase in the number of granulomas per square micron in CA-immunized mice. Thus, there are more granulomatous lesions in CA-immunized mice, but they have similar size and composition. Taken together, these data suggest that increased T cell activation, macrophage activation, and expression of chemokines does not appear to significantly alter the structure of the granuloma.

**Figure 6:**
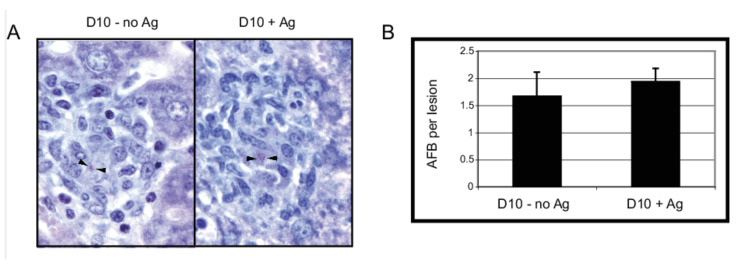
Immunization with CA does not result in dissemination or loss of bacterial control. (**A**), Formalin fixed liver tissue from nonactivated and activated mice was stained by the Ziehl–Neelsen method for acid-fast bacilli (arrows). (**B**), Number of acid-fast bacilli were counted microscopically at 1000× total magnification. Error bars represent standard error of the mean (SEM). Note that no bacteria were detected outside of lesions. Data representative of three independent experiments with 2–4 mice per group.

The number of AFB was counted on Ziehl–Neelsen stained formalin fixed liver sections (Figure 6) to determine the effect of the activation of non-specific T cells on bacterial control. Despite differences observed in macrophage activation and secretion of IFN-γ, no statistically significant differences were observed in the number of AFB per lesion. The lack of “improved” control of infection may reflect that there is already sufficient protective granuloma formation in both 5CC7 RAG^−/−^ mice and 5CC7 RAG^−/−^ mice transferred with D10 T cells even without the help of activated BCG-non-specific D10 T cells. For comparison, granulomas from wild-type B10.BR mice with a normal T cell repertoire contain on average 3 AFB per lesion while B10 RAG^−/−^ mice contain on average 23 AFB per lesion [15]. Thus, BCG infection appears to be well controlled in both 5CC7 RAG^−/−^ and 5CC7 RAG^−/−^ mice transferred with D10 T cells. These data are consistent with our previous findings that a single T cell is sufficient for protective granuloma formation and further suggest that the presence of non-specific activated T cells does not detract from protective granuloma formation and additionally is not able to enhance it any further. It also indicates that anergized T cells can induce granulomas and control bacterial expansion, and that factors other than IFN-γ can contribute to these activities.

## 4. Discussion

In chronic mycobacterial infections, mycobacteria are confined and controlled within granulomas. CD4^+^ T cells are central in granuloma formation [24,25,26]. Depletion of CD4^+^ T cells results in disorganized granuloma structure, reactivation and dissemination of infection, and eventually death of the animal. The relative role of BCG-specific and BCG-non-specific T cells in granulomas is not understood and that is the subject of this paper. In contrast to wild-type granulomas, CD4^+^ T cells in mice expressing a monoclonal population of BCG-specific T cells have a resting phenotype. We hypothesized that the activated phenotype observed in the granulomas of wild-type mice at six weeks postinfection was due to a complex T cell repertoire that includes BCG-non-specific T cells. To test this hypothesis, we adoptively transferred D10 T cells representing a BCG-non-specific T cell into this monoclonal T cell system. D10 T cells were shown by a number of criteria to be nonreactive to PCC-BCG (Figure 2, middle row, Figure 3, middle row). In this work, we show that activated non-specific T cells are able accumulate in the granuloma. In addition, these granuloma-infiltrating non-specific T cells secrete a broad range and large amount of cytokines and chemokines, can influence BCG-specific T cells at that site, and can potentially affect granuloma function. These data suggest that non-specific T cells in the context of a normal T cell repertoire may play a role in chronic granuloma formation. Finally, while D10 T cells appear to enhance the activity of granuloma-infiltrating BCG-specific T cells, they appear to suppress some functions of BCG-specific T cells at the systemic level. 

### 4.1. Accumulation of Non-Specific T Cells

Accumulation of T cells at an inflammatory site depends both on activation molecules expressed by T cells as well as on the presence of antigen at the site and its effect on retention of antigen-specific cells. Studies examining the homing of T cells to inflamed skin in the presence or absence of antigen illustrate that while T cells have access to inflammatory sites independent of antigen specificity, local antigen aids in retention of antigen-specific T cells in the absence of proliferation [27]. Similarly, effector or memory but not naïve OT-1 T cells were able to accumulate in influenza-infected lungs [28]. Studies from our lab describing the homing of a CNS-antigen-specific T cell line [29] and of LCMV-specific T cells [18] to granulomatous inflammatory sites have reached similar conclusions. The present work shows that activated non-specific T cells can not only accumulate in the granuloma, as observed previously, but can even dominate the site. In addition, the induction by activated granuloma-infiltrating non-specific T cells of the chemokines MIP-1α, MCP-1, RANTES and IP-10, which preferentially attract effector T cells [30,31], helps serve as a positive feedback loop in the recruitment of non-specific T cells at the site [32]. All these argue that the systemic activated T cell repertoire has access to inflammatory sites. Early in infection, in the presence of high levels of infectious agent, a lot of the systemic activated T cells are likely to be specific for the infectious agent. In the chronic stage when pathogen loads are lower, pathogen-specific T cells represent a smaller proportion of the systemic activated T cell repertoire. Thus, in chronic granulomas the proportion of pathogen-non-specific T cells is likely to be higher. Recent work using a systems biology simulation approach has suggested that non-specific T cells may actually compete with Mtb-specific T cells for access to infected macrophages [33]. This would seem to conflict with our data that recruitment of non-specific T cells actually increases activation of mycobacteria-specific T cells and results in increased macrophage expression of MHC class II implying activation by T cell-derived IFN-γ. One possibility is that IL-2 secreted by non-specific activated T cells directly activates local anergized specific T cells.

### 4.2. Anergy and Granuloma Function

In this study, we report that chronic infection of mice with a monoclonal BCG-specific T cell population results in an anergic state marked by downregulation of the activation marker LFA-1 as well an inability to secrete IFN-γ or IL-2 in response to restimulation with cognate antigen. In chronic mycobacterial infection, hyporesponsiveness of T cells to antigen has been reported. Peripheral blood T cells of lepromatous leprosy patients express lower levels of TCR ζ chains, p56^lck^ and NF-κB p65 correlating with decreased transcriptional activity from the IFN-γ promoter [34]. Similarly, peripheral blood T cells from TB-infected patients demonstrate lower levels of TCR ζ chains [35]. The same work demonstrated that Mtb granuloma-infiltrating T cells produce little IFN-γ when restimulated with antigen, but that IFN-γ secretion could be increased by the addition of IL-2. Similar results have been reported for synovial infiltrating T cells in rheumatoid arthritis patients [36]. Taken together, these data suggest that chronic inflammation, such as that induced by BCG infection in our system, can induce T cell anergy.

T cell hyporesponsiveness can be induced by the presence of excess antigen or by other factors in the T cell’s local microenvironment. Chronic infection of mice with LCMV results in deletion of LCMV tetramer-specific T cells [37]. In addition, there is a progressive loss of the ability of the remaining LCMV tetramer-specific T cells to secrete IL-2, TNF, and IFN-γ similar to what is observed in chronic PCC-BCG infection of 5CC7 RAG^−/−^ mice in the present work. The severity of this deletion and inactivation correlates with the level of LCMV antigens present in the mouse. Interestingly, adoptive transfer of LCMV GP-specific CD4^+^ or CD8^+^ TCR Tg T cells demonstrated that CD4^+^ T cells took 5–6 weeks to become anergized [38], similar to the timeframe seen in our model of BCG infection. In a mouse model of tolerance, a panel of hen egg lysozyme (HEL) transgenic mice was crossed to the 3A9 CD4^+^ TCR Tg mouse. In this system, the level of T cell inactivation correlated with the amount of antigen present in the lymph nodes and the affinity of the TCR-pep-MHC interaction [39]. In another system, male antigen-specific T cells become anergic when transferred into male nude mice, but this anergy can be reversed by retransferring these cells into female nude mice [40]. How this applies to the BCG model system is unclear. In our system, BCG bacterial load is high at three weeks but drops significantly by six weeks. High levels of antigen early in infection may induce T cell anergy which then persists for the duration of the experiment.

Alternatively, hyporesponsiveness of granuloma-infiltrating T cells could be the result of antigen deprivation of T cells. In BCG or Mtb granulomas, the display of bacterial antigen is very low, even in the acute phase. Overexpression of antigen by recombinant BCG activates T cells to much higher levels of cytokine production indicating that only a fraction of the host effector capacity is used [41,42]. Antigen deprivation may also occur through competition for antigen presenting cells between mycobacteria-specific T cells with non-specific T cells. In Mtb granulomas, the ratio of bacterial specific T cells is reported to be less than 5% of granulomas T cells [43]. Additionally, the granuloma structure itself in which T cells are localized to the periphery and most infected macrophages reside in the center of the granuloma might sequester T cells away from antigen presenting cells. 

Antigen-independent factors in the granuloma microenvironment may also play a role in inducing T cell anergy. Chronic exposure of T cells to TNF in vitro has been shown to cause downregulation of TCR ζ chains and hyporesponsiveness to antigen restimulation [44,45]. Sarukhan, et al. [46] demonstrated that less than half of mice doubly transgenic for a TCR specific for influenza hemagglutinin and for expression of hemagglutinin in pancreatic beta cells develop clinical diabetes. The remaining mice which do not develop diabetes have infiltration of pancreatic islets by transgenic T cells which proliferate poorly when restimulated with antigen. This hyporesponsiveness was correlated with a higher secretion of TNF by islet-infiltrating T cells, providing circumstantial evidence for a role for TNF in promoting anergy at this site. Furthermore, expression of TNF under the control of the same beta cell specific promoter prevented the development of autoimmune diabetes in the susceptible NOD mouse strain [47]. The expression of immune checkpoint molecules can also restrict granulomatous responses. Chronic granulomas possess dendritic cells expressing PD-L1 and T cells expressing PD-1 [48]. Inhibiting PD-1-PD-L1 interactions allows T cells to produce higher IFN-γ [48]. Other inhibitory receptors on T cells and regulatory cells can further limit granuloma T cell responses [25]. Taken together, these data suggest a role for inflammatory cytokines such as TNF or immune checkpoint molecules such as PD-1-PD-L1 in rendering T cells hyporesponsive during chronic inflammation.

**Figure 7:**
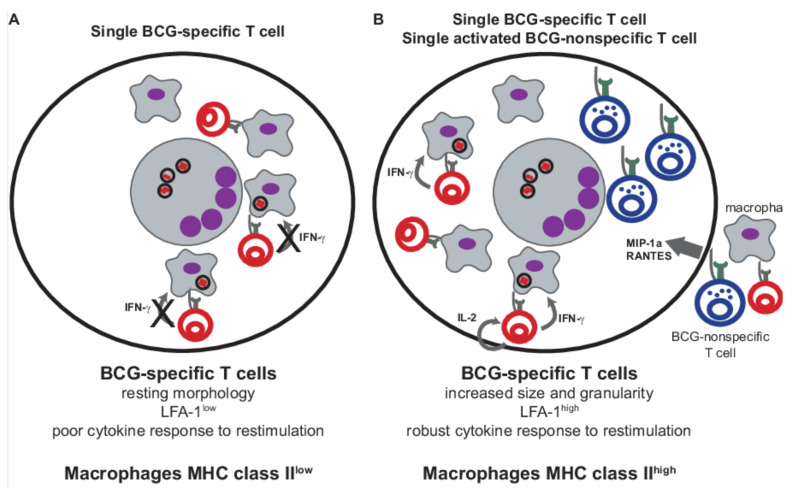
The effect of BCG-non-specific T cells on the function of BCG-specific T cells and BCG-induced granulomas. (**A**), BCG granulomas in a BCG-specific, monoclonal T cell granuloma control BCG infection despite anergic T cells with a nonactivated phenotype and poor cytokine secretion as well as low levels of macrophage activation. (**B**), An activated, BCG-non-specific T cell is able to accumulate in the granuloma and rescue the anergic BCG-specific T cells, resulting in the BCG-specific T cells acquiring an activated phenotype, increasing cytokine secretion and activating macrophages.

We report that the presence of an activated non-specific T cell population can partially reverse the hyporesponsiveness of 5CC7 T cells despite all the limiting factors discussed above. (See schematic, Figure 7) Classically, IL-2 is thought to be the most important cytokine in reversal of anergy [49]. This may be relevant in our system, since activated D10 T cells secreting IL-2 are recruited to the granuloma and in addition induce 5CC7 T cells to produce IL-2. Also, D10 T cells in our system produce a broad range of cytokines. Other cytokines such as IL-18 [50,51] and cytokine combinations such as IL-2, IL-6 and TNF [52] have been shown to induce T cell activation in the absence of antigen. Alternatively, chemokine secretion by D10 T cells (Table 1) may allow recruitment of nonanergic 5CC7 T cells from the systemic pool, which are clearly able to secrete IFN-γ and IL-2 (Table 2). Further study will clarify the mechanism by which anergy is broken.

Interestingly, despite a minimal ability to secrete Th1 cytokines, PCC-specific T cells are still able to form granulomas similar in appearance to those of wild-type mice and are also able to control bacterial numbers to similar levels (Figure 5 and Figure 6). This seems paradoxical since depletion of CD4^+^ T cells during the chronic phase of infection results in reactivation [17]. One possibility is that anergic T cells can under some conditions still produce IFN-γ [53,54]. Another possible resolution of this paradox is that factors besides the cytokines and chemokines assayed may compensate for their loss. For example, although IFN-γ and TNF are thought to be essential cytokines in protection against mycobacterial infections, IFN-γ- and TNF-independent mechanisms are known to exist for protection against *M. tuberculosis* [25,55,56]. In addition, while granuloma-infiltrating 5CC7 T cells are anergic, systemic 5CC7 T cell retain the ability to secrete cytokines and a low level of recruitment to the granuloma may be enough to provide some protection. Finally, the protection observed may be a remnant of the more active T cell response seen at earlier times during infection and that eventually BCG infection would reactivate in 5CC7 RAG^−/−^mice at later times than those tested in the present work. These data support one interpretation that anergized T cells contribute to granuloma maintenance and bacterial control and that factors other than IFN-γ may be responsible for these activities.

### 4.3. Activated D10 T Cells Suppress Systemic PCC-Specific T Cells

In contrast to the T cell cooperation observed in the granulomas of these mice, activated D10 T cells appear to inhibit cytokine secretion by 5CC7 cells in the systemic compartment as represented by the spleen. As shown in Table 1, CA-induced secretion of several cytokines, notably IFN-γ and IL-2, is correlated with a tenfold or more decrease in PCC-induced secretion of the same cytokines. Since the D10 T cells constitute only half of the T cells in the spleens of D10-transferred, CA-immunized mice, this difference cannot be explained by cell numbers alone and suggests an active mechanism of suppression in contrast to the passive competition observed between T cells for peptide-MHC complexes on antigen presenting cells [57,58,59]. Thus, while activated non-specific T cells help resting granuloma-infiltrating T cells, activated non-specific T cells compete with activated granuloma-infiltrating T cells. Suppression of IFN-γ secretion by IL-4 is observed in Th2 polarization protocols, but this seems unlikely in this system given that D10 T cells are secreting a Th1 profile of cytokines. Work by Duthoit and colleagues [60] demonstrated that recently activated CD4 T cells can suppress the proliferation of and secretion of IL-2 by naïve T cells when the two are cultured together. This effect is not diminished by addition of up to a 50:1 ratio of irradiated stimulator spleen cells to T cells and persists even after the naïve cells are separated from the activated cells arguing against competition for access to antigen presenting cells. The mechanism of suppression is not clear but appears to depend on cell-cell contact. In our system, D10 cells are acutely activated and may have similar suppressive effects on 5CC7 either in vivo or in vitro during the assay. In summary, T cells can interact to either positively or negatively regulate each other. It is clear from the data presented here that the T cell compartment (in this case, systemic vs. effector site) is one factor influencing whether the interaction is positive or negative. Further study will be necessary to determine how this occurs.

## 5. Summary

The large size of the normal T cell repertoire obscures our understanding of the basic rules governing their interactions with each other and ultimately determining the shape and character of the immune response. In this work, we employ a two T cell network of known specificities to understand how T cells interact in the context of a chronic infection. Using this system, we observed a number of interesting phenomena. Similar to previous work, activated non-specific T cells were able to accumulate in the granuloma and even dominate the site. These non-specific T cells were also able to significantly increase the activation state of and cytokine secretion by BCG-specific T cells at that site. Indeed, granuloma infiltrating BCG-specific T cells exhibited behavior consistent with anergy and the presence of activated non-specific T cells allowed a partial reversal of this state. Together with the heterogenous nature of the TCR repertoire at the single granuloma level previously observed, these data suggest that non-specific T cells may play a role in maintaining the activation state of T cells in chronic granulomatous diseases. In contrast, activation of non-specific T cells appeared to suppress cytokine secretion by systemic BCG-specific T cells. This suggests that nature of T cell interactions is highly dependent on the T cell compartment. In summary, the use of a small network of T cells of defined specificity has revealed interesting properties of T cell interactions, which would not have been evident in the context of the full T cell repertoire. Further study will be required to understand the mechanisms behind these phenomena and their significance in T cell interactions within the full wild-type repertoire.

## Figures and Tables

**Table 1:** Activated non-Specific T cells have the Potential to Produce a Diverse Set of Cytokines and Chemokines ^1^.

	D10 Transferred, CA Immunized			
	Spleen		Granuloma	
	Media	CA	Media	CA
Cytokines				
IL-2	<3.2	1894.3	<3.2	520.1
IFN-γ	<3.2	>10,000	57	>10,000
TNF	13.3	1323.2	145.1	488.9
IL-17	<3.2	61.1	3.9	246.9
IL-4	<3.2	368	<3.2	118.6
IL-5	<3.2	32.6	17.7	114.7
IL-9	<3.2	7.8	<3.2	14.2
IL-13	<3.2	414.4	<3.2	86.2
IL-1α	<3.2	172.8	9.3	215.7
IL-1ß	12.5	211	66.9	314
IL-6	83.6	>18,000	4968.2	9256.5
IL-10	<3.2	231.7	81	396.2
Chemokines				
MIP-1α	21	1401	66	>10,000
MCP-1	243	2101	2057	1536
RANTES	<3.2	70	84	179
IP-10	237.6	400	310.6	531.5
KC	<3.2	7	333	456
Macrophage Growth Factors				
GM-CSF	<3.2	7439	115	4908
G-CSF	<3.2	2034.6	858.1	1991
D10 T cells/10^6^ cells	8.55 × 10^4^		6.7 × 10^4^	

^1^ All units are pg/mL. Data representative of one experiment with 3 mice per group.

**Table 2:** Activation of non-Specific T cells Rescues the Anergic Cytokine and Chemokine Secretion Profile of Chronically Activated BCG-Specific T Cells ^1^.

	Spleen						Granuloma					
	Nontransfer		D10—No Ag		D10 + CA		Nontransfer		D10—No Ag		D10 + CA	
	Media	PCC	Media	PCC	Media	PCC	Media	PCC	Media	PCC	Media	PCC
Cytokines												
IL-2	<3.2	515.9	<3.2	363.1	<3.2	46.3	<3.2	<3.2	<3.2	<3.2	<3.2	15.9
IFN-γ	<3.2	>10,000	<3.2	>10,000	<3.2	934	<3.2	<3.2	<3.2	<3.2	57	377
TNF	6.2	455.5	7.7	494	13.3	208.9	10.7	18.6	3.9	13.5	145.1	190.5
IL-17	<3.2	32	<3.2	6.4	<3.2	9.6	<3.2	<3.2	<3.2	<3.2	3.9	72.4
IL-4	<3.2	3.7	<3.2	11	<3.2	<3.2	<3.2	<3.2	<3.2	<3.2	<3.2	<3.2
IL-5	<3.2	12.1	<3.2	10.5	<3.2	3.4	<3.2	<3.2	<3.2	<3.2	17.7	45.4
IL-9	<3.2	<3.2	6.5	6.5	<3.2	<3.2	10.1	<3.2	<3.2	<3.2	<3.2	<3.2
IL-13	<3.2	6.6	<3.2	9.5	<3.2	<3.2	<3.2	<3.2	<3.2	<3.2	<3.2	7.9
IL-1α	<3.2	114.3	<3.2	105.8	<3.2	9.6	<3.2	<3.2	<3.2	<3.2	9.3	27.4
IL-1ß	6.1	111.4	8.2	91.6	12.5	30.2	<3.2	<3.2	<3.2	<3.2	66.9	83.8
IL-6	15.2	7913.2	32.8	8930.1	83.6	2486.4	489.2	633.3	45.5	66.6	4968.2	8554.9
IL-10	<3.2	91	8.3	84.1	<3.2	53.4	<3.2	10.5	<3.2	<3.2	81	169.4
Chemokines												
MIP-1α	22	4259	34	1201	21	89	66	126	5	25	66	755
MCP-1	191	803	338	1442	243	1459	465	455	43	55	2057	2637
RANTES	<3.2	516	<3.2	379	<3.2	76	4	11	<3.2	6	84	184
IP-10	271.9	681.2	263.7	367.1	237.6	369.8	31.7	33.9	<3.2	9.4	310.6	590
KC	<3.2	9	<3.2	5	<3.2	<3.2	75	114	10	14	333	580
Macrophage Growth Factors												
GM-CSF	<3.2	762	21	1126	<3.2	422	15	21	<3.2	<3.2	115	354
G-CSF	<3.2	915.1	<3.2	1254.2	<3.2	408.5	51.9	73.5	9.7	16.4	858.1	2348.3
5CC7 T cells/10^6^ cells	3.9 × 10^5^		2.0 × 10^5^		1.2 × 10^5^		4.0 × 10^4^		4.7 × 10^4^		1.7 × 10^4^	

^1^ All units are pg/mL. Data representative of one experiment with 3 mice per group.

## Data Availability

The data presented in this study are available on request from the corresponding author.

## Supplementary Materials

The following are available online at https://www.mdpi.com/article/10.3390/cells10123285/s1, Data demonstrating the size of transgenic T cells as a measure of activation are provided in Supplemental Figure S1: Activated non-specific T cells increase the activation of BCG-specific T cells as measured by cell size. Granuloma cells were prepared from mice treated as described in the legend to Figure 3. Cells were gated on CD4 and either 5CC7 (KJ25) or D10 (3D3) transgenic T cells and analyzed for cell size.

## Abbreviations

CA	conalbumin
PCC	pigeon cytochrome C
HEL	hen egg lysozyme
BCG	*Mycobacterium bovis* strain bacille Calmette-Guérin
Mtb	*M. tuberculosis*
Tg	transgenic
AFB	acid-fast bacilli
LCMV	lymphocytic choriomeningitis virus.
